# Emergency dental visits and no-shows among children and adolescents in public oral health service in Finland: a retrospective evaluation

**DOI:** 10.2340/aos.v85.46926

**Published:** 2026-09-25

**Authors:** Shweta Goswami, Jussi Furuholm, Minna Kaila, Battsetseg Tseveenjav

**Affiliations:** a Clinicum, Faculty of Medicine, University of Helsinki, Helsinki, Finland; b Head and Neck Center, University of Helsinki and Helsinki University Hospital, Helsinki, Finland; c Institute of Dentistry, University of Turku, Turku, Finland; d Department of Maxillofacial Surgery, Päijät-Häme Central Hospital, Päijät-Häme Joint Authority for Health and Wellbeing, Lahti, Finland

**Keywords:** Emergency dental care, no-show, children, adolescents, retrospective

## Abstract

**Objective:**

To find out the occurrence of emergency visits and compare the co-existence of emergency visits and dental no-shows among children and adolescents in public oral health services in Helsinki, Finland.

**Methods:**

This was a register-based retrospective follow-up study. The data were collected from electronic health information register systems, Effica and Lifecare, of under-18-year-olds who attended public oral health care provided by the City of Helsinki during 2006–2020. The information collected for this study was patients’ age, sex, actualised, missing and emergency dental visits, as well as all actualised visits’ oral examination details and preventive, restorative and orthodontic treatment procedures (*n* = 200,790). Regression models were used to assess odds ratios (OR) and Rate ratios (RR).

**Results:**

The overall occurrence of emergency dental visits was 29.2%. It was 21.9% among individuals who had no dental no-shows and 43.7% among those who had at least one or more no-shows. No-shows were linked to 24% higher odds of emergency visits in the fully adjusted model (OR = 1.24, 95% confidence interval [CI]: 1.21–1.27, *p* < 0.001). The finding remained significant, with nearly a fivefold increase in odds among individuals receiving both orthodontic and restorative treatment (G4) (OR = 4.79, 95% CI: 4.62–4.96, *p* < 0.001) than among those receiving check-ups and preventive visits (G1) in the fully adjusted model. Temporal analysis showed that 44.5% of individuals had a no-show preceding their first emergency visit, while 55.5% had an emergency visit prior to their first no-show.

**Conclusions:**

Dental no-shows and emergency dental visits were linked, and their temporal pattern was bidirectional.

## Introduction

Emergency visits are typically regarded as unscheduled appointments linked to acute clinical issues [1]. Neglected oral health issues may advance to lesions that require emergency dental visits [2]. Patients requiring frequent emergency visits often overlook routine dental care and their underlying systemic health conditions [3]. The financial and social implications of these visits are significant for both children and their parents, as school days are lost and parents must take time off work to accompany their children to the dental clinic [4]. Moreover, the utilisation of dental care for emergency visits considerably burdens the healthcare system and its resources [5].

In Finland, children and adolescents are entitled to free-of-charge public oral health services, including orthodontics since 1972 [6]. The City of Helsinki is the biggest public oral health service provider. Emergency dental care service is organised by municipalities and provided at local dental clinics of health centres located in children’s and adolescents’ residential area [7], even beyond standard operating hours, according to the national healthcare plans [8]. When an adolescent or child’s caregiver with a need for emergency dental care calls, dental services schedule an appointment at local dental clinics during standard operating hours and refer to centralised emergency care beyond operating hours or at weekends. Thus, acute dental problems are primarily treated in the health centre’s local dental clinics (Figure 1). In case of serious problems, such as significant pain or swelling, or serious mouth or facial injury, a patient can be referred or scheduled directly to the emergency service at the hospital level [9]. Among children in two Finnish cities, the primary causes of emergency dental visits were caries and its complications (64%) [10]. Over 60% of the emergency dental visits, however, constituted true emergencies, with patients experiencing significant pain and requiring quick assistance [10]. A recent study on emergency dental care for COVID-19 patients in the hospital district of Helsinki and Uusimaa found that the primary issues necessitating emergencies were caries (66.5%) and pericoronitis (10.9%) [11].

**Figure 1:**
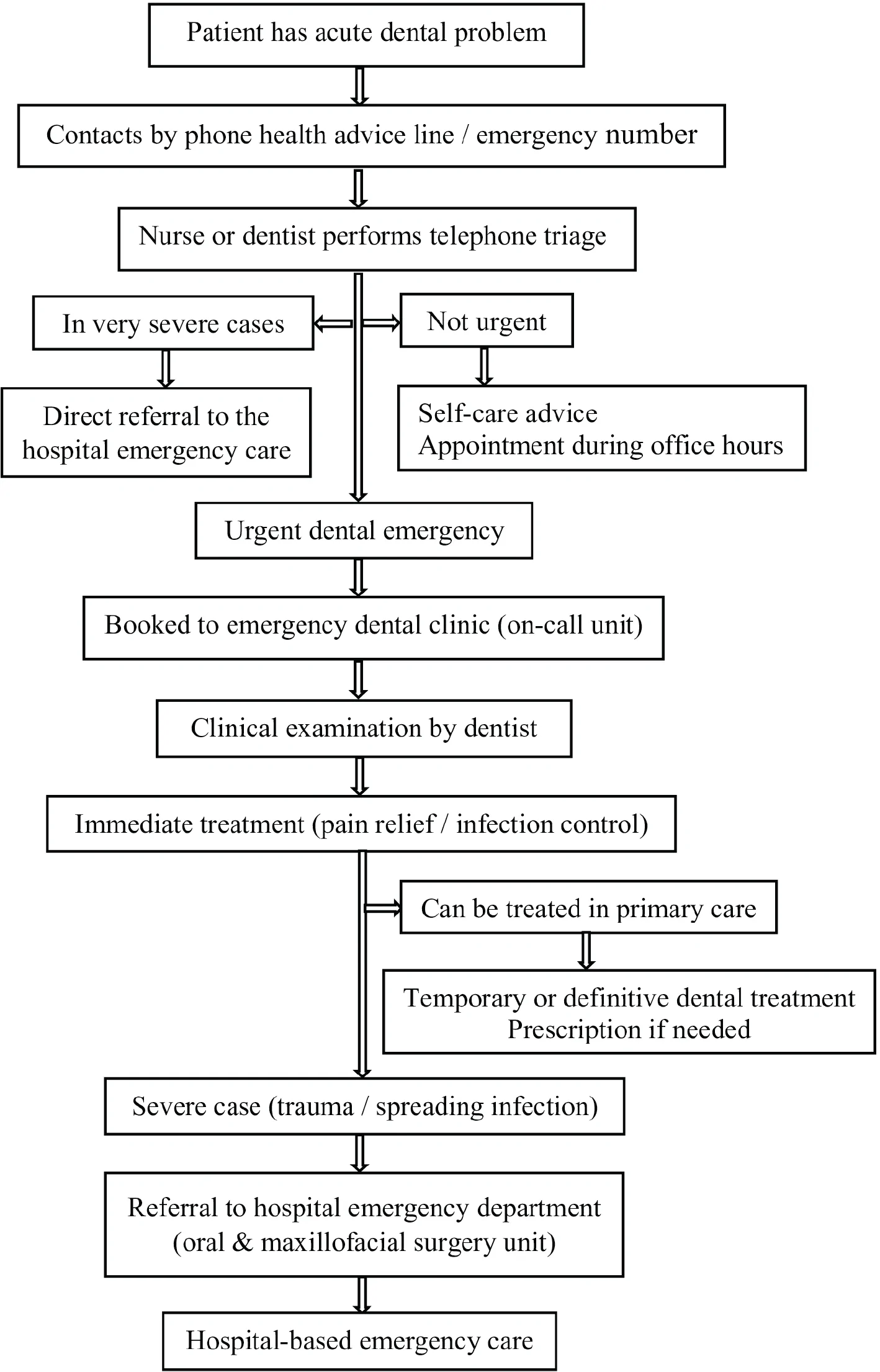
Emergency Dental Care Pathway (Primary Health Care – Finland).

Limited research has been conducted on emergency dental care at Finnish health centres [10, 12]. If oral health services are free to all children and adolescents, one might expect that the need for emergency care for dental conditions is scarce. Furthermore, it is crucial to ascertain whether individuals are neglecting regular check-ups and ultimately resorting to emergency services. It is important to analyse the trend of emergency visits for dental issues to reduce the burden on both individuals and emergency services.

To our knowledge, the frequency of dental emergency visits has not been previously investigated in this population. Therefore, the aim of this study was to determine the overall occurrence of dental emergencies and compare the co-existence of emergency visits and dental no-shows among children and adolescents under 18 years of age from 2006 to 2020.

## Materials and methods

### Study design and setting

This was a retrospective register-based study. Thus, the data were collected from patient registers of all children and adolescents treated in the public oral healthcare service of the City of Helsinki, using the EFFICA information system for years 2006–2017 and LIFECARE for years 2018–2020, allowing a 15-year follow-up. A detailed study design was described in our previous study [13]. Each patient register includes the child’s or adolescent’s actualised, missed, and emergency dental visits as well as all treatments they received during the study period, based on dentists’, dental hygienists’ or dental nurses compiled information. Thus, the numbers of children and adolescents attending the emergency services were obtained from patient attendance records covering the whole study period. Dental appointments for children and adolescents under 18 years old are scheduled by public health centres of the municipalities for both check-up examinations and treatment visits. All these visits are registered in the health information system, including instances where the individual misses the appointment without cancelling. In Finland, each patient visit type and treatment procedure is compiled using specific codes, which are dictated by the Finnish National Institute of Health and Welfare.

### Study population, further grouping and variables

A total of 200,799 individuals under 18 years of age were identified from the electronic patient information systems during the study period (2006–2020). Individuals with missing data were excluded from this study (*n* = 9), and the final sample consisted of 200,790 individuals. The information collected from the registers for this study was patients’ age, sex, actualised, missed, and emergency dental visits, as well as all actualised visits’ oral examination details and preventive, restorative and orthodontic treatment procedures.

For analysis, the study subjects were classified into two groups, according to their missed appointments without prenotification: (1) subjects with one or more no-shows or (2) subjects without no-shows. According to emergency visits, subjects were grouped into two groups: (1) subjects with one or more emergency visits or (2) subjects without emergency visits.

Scheduled appointments were recoded based on later events into: (1) actualised visit, if an oral examination or treatment code was identifiable in patient visit records, (2) no-show (missed appointment without pre-notification), if code 900, the specific code for missed appointment, was identifiable in the records, (3) emergency visit, if code 456, specific for emergency visit, was identifiable in the visit compilation.

Based on treatment procedure codes compiled in their registers during actualised visits, study subjects were grouped into four: G1 = check-ups and preventive visits, G2 = check-ups, followed by restorative procedures and/or extraction, G3 = check-ups, followed by orthodontic treatment, G4 = check-ups, followed by both G2 and G3 treatments.

### Statistical analysis

The statistical analysis was carried out using the SPSS 28.0 software (SPSS Inc., Chicago, IL, USA). A descriptive analysis was conducted to summarise baseline characteristics. Comparisons between individuals with and without no-shows were made using independent-samples t-tests and chi-square tests.

Binary logistic regression was used to assess the effect of no-shows on emergency visits, including both adjusted and unadjusted models. Odds ratio (OR) with 95% confidence intervals (CI) were calculated. The modelling of the variables was first conducted to assess the independent effect of no-shows on emergency visits. Further, the models were adjusted in a sequential way: age (Model 1A); age and sex (Model 1B); age, sex and treatment groups (Model 1C); age, sex, treatment groups and follow-up years (Model 1D). To address the potential loss of information due to dichotomisation, negative binomial regression was used to model the number of emergency visits. The logarithm of total visits was included as an offset variable. A small constant (0.01 years) was added to follow-up time to ensure non-zero exposure for all individuals. The same sequential adjustment was applied as in the binary logistic regression models (Model 2A–2D). Findings from the negative binomial models are presented as rate ratios (RR) with 95% CIs. Zero-inflated negative binomial models were explored but yielded a non-significant zero-inflation component and model instability, and therefore were not incorporated in the final analysis. Temporal precedence was determined by identifying which event occurred first – a dental no-show or a dental emergency visit.

## Results

### Study population characteristics and distribution of treatment groups

The mean age of the study population at baseline (first recorded visit) was 6.22 years (standard deviation, SD = 5.2). About half of the study population were male (50.9%). The mean follow-up time was 4.9 years (SD = 4.1). The median follow-up was 4.2 years (interquartile range: 1.1–8.4), with a range from 0.00 to 14.98 years. Overall, 29.2% of individuals had at least one emergency dental visit during the follow-up period. The frequencies of emergency visits by age, sex, follow-up year, and treatment group are presented in Table 1.

**Table 1:** Frequencies of emergency visits by age, sex, follow-up year, and treatment group in Helsinki health centres between 2006 and 2020 (N = 200,790).

Characteristics (*n* = 200,790)	Subjects with no emergency visits in their register (*n* = 142,095)	Subjects with one or more emergency visits in their register (*n* = 58,695)	*p*-value
Sex			= 0.03
Female (*n* = 98,559) Male (*n* = 102,231)	71.0%	29.0%	
	70.6%	29.4%	
Age (years), mean ± SD	6.01 ± 5.4	6.71 ± 4.5	< 0.001
Follow-up years, mean ± SD	4.02 ± 3.84	7.27 ± 4.0	< 0.001
Treatment groups according to reason for dental visits based on procedure codes			< 0.001
G1: check-ups and preventive visits	90.3%	9.7%	
G2: restorative procedures and/or extraction	61.7%	38.3%	
G3: orthodontic treatment visits	70.9%	29.1%	
G4: G2 + G3	42.6%	57.4%	

SD: standard deviation.

Emergency dental visits occurred more frequently in the no-show group (43.7%) than those without no-shows (21.9%). Overall, 33.5% (*n* = 67,283) had at least one no-show. The stratification by no-show record showed that individuals with no-shows were slightly older at baseline than those without no-shows. Males had a higher proportion in the no-show group (53%) than with without no-show group (49.8%). The distribution of treatment groups (G1–G4) varied significantly according to no-show record (Table 2).

**Table 2:** Characteristics of individuals by dental no-show record in Helsinki health centres between 2006 and 2020 (N = 200,790).

Characteristics (*n* = 200,790)	Subjects with one or more no-shows in their register (*n* = 67,283)	Subjects without no-shows in their registers (*n* = 133,507)	*p*-value
Sex			< 0.001
Female (*n* = 98,559) Male (*n* = 102,231)	47%	50.2%	
	53%	49.8%	
Age, mean ± SD	7.89 ± 4.6	5.37 ± 5.2	< 0.001
Follow-up years, mean ± SD	6.57 ± 3.91	4.16 ± 4.06	< 0.001
Emergency visits			< 0.001
No (*n* = 142,095) Yes (*n* = 58,695)	56.3%	78.1%	
	43.7%	21.9%	
Treatment groups according to the reason for dental visits based on procedure codes			< 0.001
G1: check-ups and preventive visits G2: restorative procedures and/or extraction G3: orthodontic treatment visits G4: G2 + G3	18.2%	55.9%	
	35.5%	23.6%	
	8.7%	7.6%	
	37.6%	12.8%	

SD: standard deviation; G: treatment group.

There were statistically significant differences observed among the groups (G1–G4) in age and follow-up duration (days and years) (*p* < 0.001). G4 demonstrated the longest follow-up duration (8.4 years) among all groups. The proportion of emergency visits varied significantly across groups (*p* < 0.001), with G4 exhibiting the highest frequency (57.4%). G4 also had a higher proportion of missed appointments than other groups (59.6%). The distribution of sex varied among groups (*p* < 0.001), with G4 having slightly more females (51.2%) than males (48.8%). G4 were more likely to be included in the longer follow-up group (>5 years) (Table 3).

**Table 3:** Characteristics of individuals by treatment group (G1–G4) between 2006 and 2020 (N = 200,790).

Characteristics (*n* = 200,790)	G1 (check-ups or examinations and preventive visits; *n* = 86,919)	G2 (restorative procedures and/or extraction; *n* = 55,409)	G3 (orthodontic treatment visits; *n* = 16,025)	G4 (G2 + G3; *n* = 42,437)	*p*-value
Age (mean ± SD)	4.6 (± 5.2)	8.2 (± 5.1)	6.6 (± 5.2)	6.7 (± 4.2)	< 0.001
Follow-up years (mean ± SD)	2.6 (± 3.2)	5.5 (± 3.9)	6.6 (± 3.8)	8.4 (± 3.4)	< 0.001
Follow-up days (mean ± SD)	969.9 (± 1161.9)	2013.9 (± 1440.1)	2427.3 (± 1401.2)	3057.5 (± 1257.6)	< 0.001
Sex					< 0.001
Female Male	48.6%	48.2%	49.0%	51.2%	
	51.4%	51.8%	51.0%	48.8%	
Emergency visits	9.7%	38.3%	29.1%	57.4%	< 0.001
No-show	14.1%	43.1%	36.4%	59.6%	< 0.001
Follow-up groups					< 0.001
< 3 years 3–5 years > 5 years	65.7%	33.2%	22.8%	8.3%	
	14.2%	15.6%	12.6%	10.7%	
	20.1%	51.3%	64.6%	80.9%	

SD: standard deviation.

Emergency visits ranged from 3.8% in G1 to 53.6% in G4. Those who received both restorative and orthodontic treatments (G4) had the highest rate of emergency visits among no-shows (53.6%) (Table 4). The distribution of no-shows across all treatment groups, stratified by emergency visits, is presented in Figure 2.

**Table 4:** Distribution of emergency dental visits by procedure type and attendance status of children with and without no-shows in Helsinki health centres between 2006 and 2020 (N = 200,790).

Treatment groups (G) (*n* = 200,790)	Visits of individuals without no- shows (*n* = 133,507) (66.5%)		Visits of individuals with no-shows (*n* = 67,283) (33.5%)	
	No emergency visit (*n* = 104,241) (73.4%)	Emergency visit (*n* = 29,266) (49.9%)	No emergency visit (*n* = 37,854) (26.6%)	Emergency visit (*n* = 29,429) (50.1%)
G1: check-ups and preventive visits (*n* = 86,919)	64.5%	25.1%	29.4%	3.8%
G2: restorative procedures and/or extraction (*n* = 55,409)	19.9%	36.9%	35.6%	35.3%
G3: orthodontic treatment visits (*n* = 16,025)	7.4%	8.6%	9.8%	7.3%
G4: G2 + G3 (*n* = 42,437)	8.2%	29.4%	25.2%	53.6%

Columns are structured by no-show group, with emergency visit status (yes/no) presented within each group.

**Figure 2:**
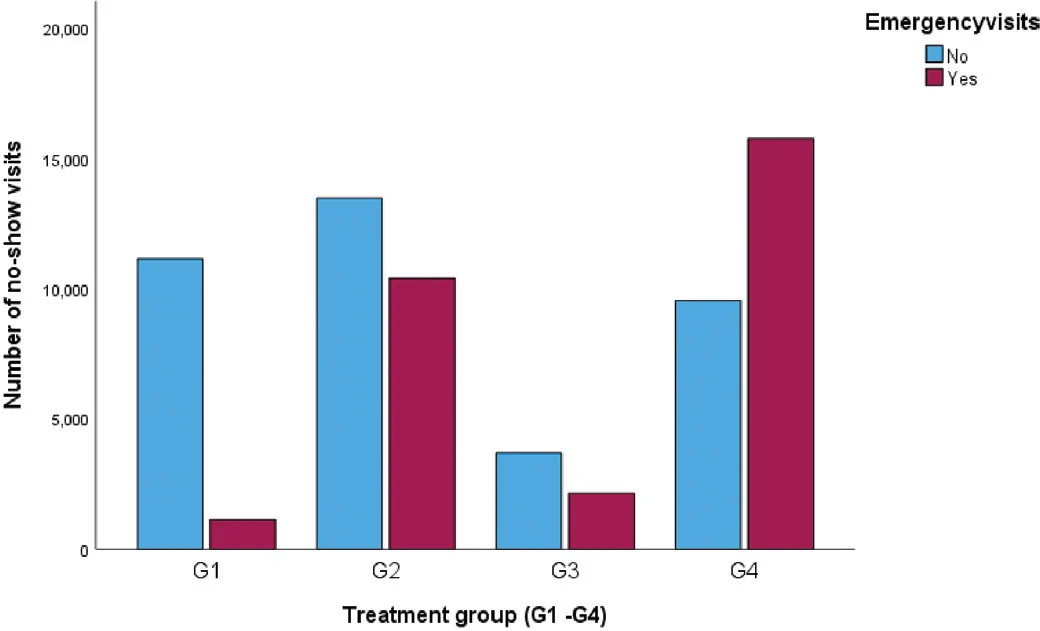
Emergency visits among children with dental no-shows across treatment groups (G1–G4) in Helsinki health centres between 2006 and 2020. G1 = check-ups and preventive visits, G2 = check-ups, followed by restorative procedures and/or extraction, G3 = check-ups, followed by orthodontic treatment, G4 = check-ups, followed by both G2 and G3 treatments

### Comparison of emergency visits with and without dental no-shows

The unadjusted logistic regression showed that children and adolescents with no-shows had 2.77 times higher odds of emergency visits than those without no-shows (95% CI: 2.71–2.83, *p* < 0.001). After adjusting for age, the effect had reduced slightly but remained significant (OR = 2.74, 95% CI: 2.68–2.80, *p* < 0.001). Further, adjusting for sex showed a similar effect to adjusting for age (OR = 2.74, 95% CI: 2.68–2.80, *p* < 0.001). In the fully adjusted model, which includes age, sex, treatment groups, and follow-up years, the magnitude of the effect had attenuated but remained significant (OR = 1.24, 95% CI: 1.21–1.27, *p* < 0.001).

Age (OR = 1.054, 95% CI: 1.051–1.057, *p* < 0.001) and sex (OR = 1.05, 95% CI: 1.03–1.07) showed minimal impact but a positive linkage with emergency visits in the fully adjusted model. In contrast, treatment groups were strongly linked with emergency visits. In fully adjusted model, G2 had approximately a threefold higher odds (OR = 3.15, 95% CI: 3.06–3.26, *p* < 0.001), G3 had nearly a twofold higher odds (OR = 1.91, 95% CI: 1.83–2.00, *p* < 0.001), and G4 had the highest odds with almost fivefold increase (OR = 4.79, 95% CI: 4.62–4.96, *p* < 0.001). Every subsequent year of observation enhanced the likelihood of emergency visits by around 17% (OR = 1.17, 95% CI: 1.16–1.17, *p* < 0.001) (Table 5).

**Table 5:** Logistic Regression predicting emergency visits by no-shows, adjusted for age, sex, and treatment groups (G).

Predictor	Crude OR (95% CI)	Model 1A (95% CI)	Model 1B (95% CI)	Model 1C (95% CI)	Model 1D (95% CI)
Having no-show (Yes vs. No)	2.769 (2.714–2.825)*	2.740 (2.684–2.797)*	2.740 (2.684–2.797)*	1.514 (1.480–1.549)*	1.241 (1.212–1.270)*
Age (per year)	–	1.004 (1.002–1.006)*	1.004 (1.002–1.006)*	0.978 (0.975–0.980)*	1.054 (1.051–1.057)*
Sex (Male vs. Female)	–	–	0.991 (0.972–1.011)	1.043 (1.021–1.066)*	1.051 (1.028–1.074)*
Treatment groups (G)					
G2: restorative procedures and/or extraction	–	–	–	5.545 (5.382–5.714)*	3.154 (3.055–0.257)*
G3: orthodontic treatment visits	–	–	–	3.638 (3.490–3.791)*	1.912 (1.829–1.998)*
G4: G2 + G3	–	–	–	11.019 (10.679–11.369)*	4.786 (4.617–4.962)*
Follow-up years	–	–	–	–	1.168 (1.163–1.172)*

Model 1A: adjusted for age; Model 1B: adjusted for age, sex; Model 1C: adjusted for age, sex and treatment groups; Model 1D: adjusted for age, sex, treatment groups and follow-up years.

Reference categories: No-shows = ‘No’; Sex = ‘Female’; Treatment group = ‘G1: check-ups and preventive visits’.

OR: odds ratio; CI: confidence interval.

* *p* < 0.001.

In negative binomial regression models, no-shows were significantly linked with higher emergency visit rates in the crude analysis (RR: 1.64, 95% CI: 1.62–1.67, *p* < 0.001). The findings remained statistically significant following adjustment for age, treatment group, and follow-up duration, though the magnitude of effect attenuated in the fully adjusted model (RR: 1.12, 95% CI: 1.10–1.14, *p* < 0.001). Sex was not statistically significant for emergency visit rates. The treatment group showed the strongest effect on emergency visit rates, with G4 individuals demonstrating consistently higher rates than other groups (RR: 2.35, 95% CI: 2.28–2.42, *p* < 0.001) (Table 6).

**Table 6:** Negative binomial regression models of emergency visit rates with offset for total visits.

Predictor	Crude RR (95% CI)	Model 2A (95% CI)	Model 2B (95% CI)	Model 2C (95% CI)	Model 2D (95% CI)
Having no-show (Yes vs. No)	1.641 (1.616–1.667)*	1.625 (1.600–1.651)*	1.626 (1.601–1.651)*	1.231 (1.210–1.252)*	1.122 (1.103–1.142)*
Age (per year)	–	1.005 (1.004–1.007)*	1.005 (1.004–1.007)*	0.995 (0.994–0.997)*	1.040 (1.037–1.042)*
Sex (Male vs. Female)	–	–	0.994 (0.979–1.009)	1.019 (1.003–1.035)	1.024 (1.008–1.041)
Treatment groups (G)					
G2: restorative procedures and/or extraction	–	–	–	2.762 (2.692–2.835)*	2.076 (2.019–2.135)*
G3: orthodontic treatment visits	–	–	–	1.973 (1.906–2.042)*	1.427 (1.375–1.480)*
G4: G2 + G3	–	–	–	3.570 (3.479–3.663)*	2.350 (2.280–2.421)*
Follow-up years	–	–	–	–	1.083 (1.080–1.086)*

Model 2A: adjusted for age; Model 2B: adjusted for age, sex; Model 2C: adjusted for age, sex and treatment groups; Model 2D: adjusted for age, sex, treatment groups and follow-up years.

Reference categories: No-shows = ‘No’; Sex = ‘Female’; Treatment group = ‘G1: check-ups and preventive visits’.

RR: rate ratio; CI: confidence interval.

* *p* < 0.001.

Stratified analysis of treatment groups showed that the effect of no-shows on emergency visit rates differed across treatment groups. No-shows were linked to lower emergency visit rates in G1 (RR: 0.64, 95% CI: 0.60–0.69, *p* < 0.001), whereas increased emergency visit rates were observed in groups with treatment needs – G2 (RR: 1.15, 95% CI: 1.12–1.18, *p* < 0.001), G3 (RR: 1.21, 95% CI: 1.14–1.28, *p* < 0.001), and G4 (RR: 1.16, 95% CI: 1.13–1.19, *p* < 0.001).

### Direction of events between no-shows and emergency visits

Temporal sequence analysis showed that among children and adolescents with both events (*n* = 29,429), 44.5% had a no-show preceding their first emergency visit, while 55.5% had an emergency visit prior to their first no-show. In addition, within treatment groups, G1 – 39% had no-show before an emergency and 61% had an emergency before no-show, whereas in G4 – 43.8% had no-show before an emergency and 56.2% had an emergency before no-show.

### Sensitivity analysis

A sensitivity analysis restricted to individuals with 10 years or less of follow-up showed that no-shows remained linked to emergency visits in the fully adjusted model (OR = 1.24, 95% CI: 1.21–1.28, *p* < 0.001). Age and follow-up duration differed significantly across treatment groups (*p* < 0.001), with large effect sizes (η² = 0.141 and 0.237, respectively). Age increased progressively from G1 (youngest) to G2 (oldest). The follow-up duration showed G4 had the longest mean follow-up (6.38 years), followed by G3 and G2, while G1 had the shortest.

## Discussion

This retrospective study assessed the occurrence of emergency visits among children and adolescents and compared emergency visits with and without no-shows among children and adolescents in public oral health services in Helsinki, Finland. The findings suggested a significant linkage between missed appointments and emergency dental visits. The likelihood of emergency dental visits increases with individuals undergoing orthodontic and restorative treatments.

The primary dental conditions, namely toothache, infection, and dental caries, are likely due to poor dental self-care and inadequate regular dental examinations. They can be prevented by screening, early intervention, and education regarding proper oral hygiene practices [14]. Previous studies have documented the utilisation of dental services for avoidable dental problems [15, 16]. This study’s findings are in accordance with the previous study suggesting missed appointments increase the likelihood of emergency dental visits [15].

Age was significantly linked with emergency visits, but the relatively small increase in odds indicates limited clinical relevance. However, this may reflect the progressive nature of dental problems, demonstrating how untreated caries advance over time and ultimately lead to dental emergencies. This may also suggest a tendency among parents to delay the treatment until an emergency occurs. Higher utilisation of the emergency department by parents is linked to increased emergency visits among their children. This suggests how parental approach around seeking emergency care may directly impact children ending up in the emergency [17].

This study has shown that males had a slightly higher proportion of emergency visits than females. A recent review conducted among patients aged 0–25 years of age showed that more than half of paediatric emergency visits are for dental conditions, namely dental caries, followed by periapical abscess, facial cellulitis, gingival conditions, and oral lesions. Most affected were children aged 0–5 years, with males being predominant [18]. According to a study, male patients required more frequent emergency dental visits and incurred more medical expenses than females, suggesting that males are generally less aware of their oral health [19].

No-shows were linked to lower emergency visit rates in G1 (RR = 0.64). This might reflect the minimal treatment needs of this group. Children attending only check-ups and preventive visits tend to have better oral health than those necessitating restorative or orthodontic treatment. Therefore, missed appointments in this group may be less likely to lead to dental emergencies.

Children and adolescents who received both orthodontic and restorative care were likely to constitute a vulnerable group. The attenuation of the odds from 11-fold to 4.79 in G4 showed that a substantial portion of the elevated risk is explained by their longer follow-up duration. However, the persistence of a nearly five-fold risk in an adjusted model suggests that treatment complexity itself plays an important role. Children and adolescents receiving both restorative and orthodontic treatment might have a higher burden of oral disease, require frequent dental visits, and might face a higher risk of treatment-related complications, which might increase the likelihood of emergency dental visits.

Negative binomial regression analysis showed G4 individuals had significantly higher rates of emergency visits, with a Model 2C RR of 3.57 that attenuated to 2.35 in the fully adjusted model. The robustness of this study is supported by agreement of the findings from two different modelling approaches with binary logistic regression and negative binomial regression. These findings suggest that treatment complexity and underlying dental conditions might be stronger determinants than no-shows alone. The longer follow-up duration observed in G4 indicates that these individuals remain within the system for prolonged periods that might allow both restorative and orthodontic procedures to accumulate. The prolonged follow-up period likely accounts for the elevated unadjusted risk and may contribute to immortal time bias.

A sensitivity analysis restricted to individuals with follow-up of 10 years or less showed consistent results, supporting the robustness of these findings. Also, the stratified analysis of treatment groups suggests the clinical consequences of no-shows have more impact on individuals with ongoing treatment needs than individuals with check-up or preventive visits.

Patients having high caries risk during their initial assessment are more prone to develop caries during the course of orthodontic treatment [20]. Therefore, caries risk should be assessed, and active lesions should be treated before the commencement of orthodontic treatment. While no-shows were significantly linked with emergency visits, underlying clinical complexity was also associated with a stronger effect on emergency visits than no-shows. Strategies targeting early identification of individuals with high treatment needs before the accumulation of restorative and orthodontic needs might give a greater reduction in emergency visits rather than only no-shows focused strategies.

A previous register-based study from the same setting found that the overall trend of dental no-shows decreased over a 15-year period. There was a 64% decrease in the number of no-shows among girls and a 67% decrease among males compared with 2006. Year-specific analysis showed significant differences in 2020, with no emergency visits and a reduced no-show proportion (10.1%) compared with other study years (34.1%). These changes may be attributable to pandemic-related changes in service organisation and how individuals sought dental care [13]. Sensitivity analysis excluding data from 2020 had similar findings to the primary analysis. G4 had the highest proportion of emergency visits, no-shows, and the longest follow-up duration. The pattern of differences across groups remained unchanged. Missed appointments were highly polarised, with approximately 5% of children and adolescents accounting for more than one-fifth of all missed appointments [13]. This small subgroup may be at higher risk, necessitating more investigation to better understand any underlying social or behavioural reasons.

Temporal sequence analysis showed no directional pattern between no-shows and emergency visits. The main results suggest that no-shows are linked to higher emergency visits; the temporal findings indicate that the link between no-shows and emergency visits is likely bidirectional instead of causal. Since the sequence is not clearly directional, no-shows should not be interpreted as a direct cause of emergency visits. From a clinical point of view, children having emergency visits might be at increased risk of future no-show. Similarly, children with frequent no-shows might be at high risk for emergency care. One possible explanation for emergency visits preceding no-shows is that emergency dental treatment often involves pain, anxiety, and distress, which may increase dental fear and make children less likely to attend further scheduled appointments. These findings underscore the importance of early identification and timely intervention in children with irregular attendance or acute dental conditions.

Child neglect is also a serious concern, as children are one of the most vulnerable population groups. Individuals who have been neglected as children are more likely to develop negative adult health outcomes such as pulmonary disease, diabetes, and oral health–related conditions [21]. Improving parental awareness and preventive education is necessary to address child dental neglect. Enhanced professional training, interdisciplinary cooperation and well-defined clinical guidelines are required for optimal diagnosis and management [22].

Finnish Current Care guidelines for caries management focus on preventive management of caries in deciduous teeth. Children need special care in caries management since their teeth are developing and they are building lifelong health behaviours. Parents have an important role in exhibiting and promoting good oral health practices among children. Fluoride toothbrushing twice daily, nutritional counselling and early clinical preventative measures are indicated, with follow-up intervals based on individual risk [23]. These recommendations are intended for preventive measures. Delayed or missed dental appointments may hinder effective caries management and increase the likelihood of emergency visits.

Dental emergencies represent a significant burden on health systems. The present study showed that emergency dental visits were lower in those attending preventive dental visits. Preventive strategies such as regular oral hygiene practices, parental awareness, and focus on high-risk individuals could reduce dental emergency visits and burden on public oral health resources.

Though Finland provides free-of-charge oral health services for children and adolescents, no-shows and emergency visits still persist. This indicates that subsidised oral healthcare alone is not enough to prevent no-shows and dental emergencies. Therefore, consistent monitoring of both no-shows and emergency visits may help clinicians identify high-risk individuals on time and facilitate timely assessment and care. Also, it is crucial to investigate underlying issues, such as sociodemographic factors, parental factors, and care-seeking behaviour, that allow the progression of dental conditions.

### Strengths and limitations of the study

The large population-based dataset of more than 200,000 children improves the reliability of the findings. The data were collected from the public health sector, which provides dental services to all children; the findings can be considered generalisable.

This study has limitations. This is a register-based study, so any documentation error made remains uncorrected. It focused on the pattern of attendance, sex, age, follow-up years, and emergency dental visits. Variables such as socio-economic background, urban/rural residence, clustering by family and geographical area, and parental factors were not included, which limits the ability to explore why children missed appointments and sought emergency care. No-shows and emergency visits were defined as binary variables; thus, individuals with more scheduled appointments may have had more opportunity to miss visits. Also, the dataset used in this study included a single procedure code (code 456) to classify visits as emergency (yes/no) without a detailed description of the reasons for emergency visits.

The classification into treatment groups, specifically G4, may introduce immortal time bias, as these individuals needed longer follow-up. Although follow-up time was adjusted and a sensitivity analysis was performed on individuals with follow-up of 10 years or less, this does not fully remove this bias. Also, individuals who relocated or exited the system during follow-up may have introduced selection bias.

## Conclusion

Dental no-shows and emergency visits were linked, particularly among those receiving both orthodontic and restorative treatment. However, the temporal pattern between dental no-shows and emergency visits appeared to be bidirectional.

From a practical standpoint, identifying individuals in time who show a repeated pattern of missed appointments and emergency dental visits could mean better continuity of care and less burden on the oral health care system.

## Declarations

### Data availability statement

The datasets used and analysed during the current study are available from the corresponding author on reasonable request.
